# Application of 18F-PSMA-1007 PET/MR Imaging in Early Biochemical Recurrence of Prostate Cancer: Results of a Prospective Study of 60 Patients with Very Low PSA Levels ≤ 0.5 ng/mL

**DOI:** 10.3390/cancers15164185

**Published:** 2023-08-20

**Authors:** Małgorzata Mojsak, Piotr Szumowski, Anna Amelian, Marcin Hladunski, Bożena Kubas, Janusz Myśliwiec, Jan Kochanowicz, Marcin Moniuszko

**Affiliations:** 1 Independent Laboratory of Molecular Imaging, Medical University of Bialystok, 15-276 Bialystok, Poland; a.amelian88@gmail.com (A.A.);; 2 Department of Nuclear Medicine, Medical University of Bialystok, 15-276 Bialystok, Poland; piotr.szumowski@umb.edu.pl (P.S.);; 3 Department of Radiology, Medical University of Bialystok, 15-276 Bialystok, Poland; 4 Department of Neurology, Medical University of Bialystok, 15-276 Bialystok, Poland; 5 Department of Regenerative Medicine and Immune Regulation, Medical University of Bialystok, 15-276 Bialystok, Poland; marcin.moniuszko@umb.edu.pl; 6 Clinical Department of Allergic and Internal Diseases, Medical University of Bialystok, 15-276 Bialystok, Poland

**Keywords:** 18F-PSMA-1007, PET/MR, prostate cancer

## Abstract

**Simple Summary:**

PSMA PET is a relatively new method of molecular imaging in patients with prostate cancer, but in just a few years it has become the basic tool and standard of diagnosis in patients qualified for radical treatment as well as in biochemical recurrence. New data regarding new PSMA ligands labeled with various radioisotopes are constantly being reported. One should also not forget about the potential benefits of PET/MR prostate cancer diagnosis. The aim of the study was to assess the potential role of 18F-PSMA-1007 PET/MR in the diagnosis of patients with biochemical recurrence of prostate cancer.

**Abstract:**

The use of 18F-PSMA-1007 and the role of PET/MR in the diagnosis of prostate cancer are not conclusively confirmed. There are reports indicating the potential pros and cons of using 18F-PSMA-1007 as well as the PET/MR technique in prostate cancer recurrence, but they are not yet included in the EAU guidelines. The aim of the study was to assess the effectiveness of 18F-PSMA-1007 PET/MR in detecting BCR lesions at very low PSA levels <0.5 ng/mL. Methods: Sixty patients with BCR after radical prostatectomy (RP) with PSA ranged 0.1–0.5 ng/mL were enrolled in a prospective study. All patients underwent simultaneous whole-body and pelvic 18F-PSMA-1007 PET/MR. The obtained results were verified by 12-month follow-up. Results: Fifty-three lesions were detected in 45 patients with 75% detection rate. The mean PSA value was 0.31 ng/mL. Of all PSMA-positive foci, 91% were localized in the pelvis, and only 9% of lesions were located in the extrapelvic region. Local recurrences were detected in 29%, PSMA-positive lymph nodes were detected in 64% of patients and bone metastases lesions were detected in 7% of patients. Conclusions: 18F-PSMA-1007 PET/MR seems to be an excellent diagnostic tool in patients with early BCR with very low PSA levels, especially with dt PSA < 6 months. The synergistic effect of combining 18F-PSMA-1007 and whole-body PET/MR with precise multiparametric assessment of pelvic lesions is of particular benefit in early BCR.

## 1. Introduction

Prostate cancer (PC) is the most common malignancy in the male population in Europe and the second in the world. Despite the constant development of early detection techniques, as well as advanced methods of local and systemic therapy, PC is the third most common cause of death due to oncological diseases in the world [1]. In recent years, the prevalence of PC continues to increase [1]. In addition, in many cases, PC becomes a chronic disease and affects various aspects of the patient’s life, leading to a decrease in the quality of life and social functioning [2].

Unfortunately, after curative-intent treatment (prostatectomy or radical radiotherapy), biochemical recurrence (BCR) occurs in 20–50% of patients [3,4,5].

Early and precise localization of the recurrence site and restaging are crucial in starting effective therapy. That enables the optimization of therapy, which significantly extends survival time, but also improves the quality of life, creating the opportunity to return to social life [4].

In the majority of patients with BCR, it is possible to radicalize the therapy or perform other effective treatment only if recurrence is detected at an early stage [4,6,7,8].

Until recently, the basic tools for recurrence localization were MRI of the pelvis, choline or acetate-PET/CT and bone scan. The abovementioned imaging techniques, due to relatively low sensitivity, give a satisfactory diagnostic accuracy first at PSA values above 1–2 ng/mL [3,9,10].

Already in the 1990s, there appeared the very first reports about the potential use of highly specific ligands that are inhibitors of prostate receptor-specific membrane antigen (PSMA) in the diagnosis and therapy of PC [11,12,13].

68Ga-PSMA-11 PET/CT has revolutionized the diagnostics of PC, both in the staging of primary lesions and in BCR. Due to its high sensitivity and specificity, 68Ga-PSMA PET/CT has become the standard of care [3,4,14,15,16,17].

In the meantime, many radiochemistry laboratories made efforts to create new PSMA ligands [18,19,20,21]. Until now, many new radiotracers have been introduced into diagnostics, also fluorinated, for example: 18F-PSMA-1007, 18F-DCFPyl or 18F-rhPSMA. The diagnostic value of 68Ga-PSMA-11 and 18F-PSMA-1007 seems to be comparable, but due to the use of different ligands and different nuclides, they differ in terms of physical properties, the radioisotopes production method, their biodistribution, and finally the excretion way and elimination from organism. Numerous reports suggest that fluorinated ligands will be the basic tracer used in the future, due to the increasing demand for PSMA PET diagnostics and the higher efficiency of cyclotron (Fluorine-18) versus generator (Gallium-68) production [20,21,22]. The advantages of 18F-PSMA-1007 coming from its elimination from the body by the hepatobiliary system are very important, and it results in minimal retention in the bladder and urethra, which are located close to the postoperative bed [20,23,24]. This could be crucial in the detection of local recurrence. Few studies suggest a potentially higher sensitivity of the 18F-PSMA-1007 technique compared to 68Ga-PSMA-11 in the detection of local recurrence [21].

In addition, Fluorine-18 has a higher energy and a longer half-life, which may have a beneficial effect on the resolution of the PET technique and enable delayed PET acquisition [24].

However, with the increase in examination and studies performed, there has appeared a suggestion of an increased risk of false-positive results for skeletal metastases, as 18F-PSMA-1007 may show nonspecific uptake in benign skeletal lesions (NSBL) [25,26].

Independently of the development of new PSMA ligands, the development of PET modality was constantly evolving. In 2011, a simultaneous PET/MR scanner was introduced for diagnostics. PET/MR, like PET/CT, belongs to hybrid imaging techniques, with the CT modality replaced by the MR technique. The PET/MR modality is still an innovative technique. International boards of specialists are working on creating a list of key applications. The usage of numerous MR images and sequences simultaneously performed and fused with PET images enables a multiparametric assessment of tissues and organs of the whole body in terms of morphology, function and metabolism. Available reports comparing the value of PET/CT and PET/MR in the diagnosis of BCR indicate the superiority of the PET/MR technique, especially in the assessment of local recurrence and involvement of regional lymph nodes [27,28]. Some authors point to the potential benefits of PSMA PET/MR examinations already at PSA < 0.2 [27].

Summarizing, both 18F-PSMA-1007 as a radiotracer and PET/MR as an imaging technique may have a high diagnostic value, especially in the assessment of local recurrence or involvement of regional lymph nodes. Additionally, there is a suggestion of potential benefits of application of the 18F-PSMA-1007 and PET/MR technique, independently, in early BCR. There are only very few reports on the clinical value of the 18F-PSMA-1007 PET/MR examination in BCR. 

Therefore, the aim of our study was to assess the diagnostic value of 18F-PSMA PET/MR in patients with early BCR at the very low level of PSA < 0.5 ng/mL.

## 2. Materials and Methods

The prospective study included 60 patients with PC after radical prostatectomy (RP) or RP with radiotherapy of the postoperative bed/pelvis, in whom early BCR was diagnosed. Serum PSA values ranged from 0.1 to 0.5 ng/mL. In addition, the dtPSA value was calculated using the method of Khan et al. [29], assessed on least two PSA measurements performed in the last 6 months.

Up to 3 months (median 32 days) prior to study inclusion, all patients underwent multiparametric MR (mpMR) of the pelvis. None of the reports’ results confirmed the presence of recurrence loci. 

Patients were qualified for the study regardless of the stage of the lesions found in the histopathological examination of the postoperative material.

The criteria for exclusion from the study were the use of hormone therapy or chemotherapy, the presence of another malignant neoplasm, the presence of metal foreign bodies constituting a contraindication for MRI, claustrophobia and body weight that made it impossible to perform the examination due to the diameter of the gantry of the scanner.

The whole-body examination (from the top of the head to the mid-thighs) was performed using the simultaneous PET/MR technique with a Biograph mMR 3 T scanner (Siemens) 80 ± 10 min after intravenous administration of 18F-PSMA-1007 with an activity of 4 MBq per 1 kilo of body weight. PET acquisition time was 3 min/bed. Whole-body MRI was performed with T1_dixon, T2_haste and T2_haste_stir images. In addition, immediately after the whole-body examination, the patients underwent a PET scan of the pelvis simultaneously with mpMR of this region. Pelvis mpMR was performed according to the Pi-RADS v.2.1 guidelines with simultaneous PET acquisition lasting 10 min [30].

The preparation of 18F-PSMA-1007 was performed according to the European Pharmacopoeia in Good Manufacturing Practice (GMP) standard [31] with ligand provided by ABX. The prerequisite for the admission of the radiotracer to be administered to the patient was the radiochemical purity of >95%.

The obtained PET/MR images of the whole body and PET/MR images of the pelvis were evaluated by two specialists: a nuclear medicine specialist and a radiologist, both with at least 5 years of experience in PET and MR diagnostics, respectively. Both specialists assessed the received images together and prepared conclusions in cooperation.

The study reports were prepared in accordance with the EANM standardized reporting guidelines v1.0 for PSMA-PET and PSMA-RADS [32,33]. 

Only lesions in categories 4 (high focal PSMA uptake at sites typical for PC with no typical lesions in CT/MR) and 5 (high focal PSMA uptake with definite anatomic confirmation) according to PSMA-RADS were taken as PC foci, which were additionally verified during the 12-month follow-up of patients, considering changes in PSA levels after targeted therapy, and the results of additional tests included FNAB (fine-needle aspiration biopsy) of one PSMA-positive extrapelvic lymph node and imaging examinations (PSMA PET, dedicated MR) to assess further changes of detected lesions. Histopathological verification of pelvic loci was not performed.

Statistical analysis of the obtained results was carried out using Statistica 13 (Statsoft) software. The nonparametric Mann–Whitney U test was used to assess the dependence of positive PSMA-PET results on PSA concentration. The Spearman’s rank-order correlation was used to correlate the PSA level with SUVmax in detected lesions. The study of the relationship between dtPSA and positive PET-PSMA was carried out using the nonparametric Pearson chi-square test of independence. In order to compare three dtPSA value ranges and SUVmax of detected lesions, in the absence of normal distribution, the nonparametric Kruskal–Wallis test was used together with the post hoc test of multiple comparisons of average ranks for all samples.

The project was financed using the MUB’s own funds.

Before starting the project, it received a positive opinion from the local bioethics committee. All patients provided written and oral consent to participate in the study.

## 3. Results

18F-PSMA-1007 PET/MR examination was positive in 45 patients with a detection rate of 75% per patient. In total, 53 lesions were detected (Table 1).

Of all the PSMA-positive malignant foci, 48 (91%) in 39 patients were localized in the pelvis (prostatic bed and lymph nodes), and only 9% of lesions were located outside the pelvis (Table 2). 

The PSA level was in the range of 0.116–0.5 ng/mL, and the mean PSA value was 0.31 ng/mL.

Generally, patients with a positive PET result have, on average, a higher PSA measurement (median is 0.340 ng/mL) than patients with a negative result (median is 0.213 ng/mL); however, these differences are not statistically significant (at the level of *p* = 0.05). It seems that if the group with a negative PET result was larger, with the same PSA measurements, it would be possible to show differences.

There was no statistically significant correlation between PSA levels and SUVmax of detected lesions (R = 0.125; *p* = 0.41).

It should be noted that nine patients had a PSA < 0.2 ng/mL (with dtPSA ≤ 6 months). In this group, recurrence foci were detected in eight patients, which is 89% of patients with a PSA level < 0.2 ng/mL.

Overall, in the whole study group, there was a statistically significant relationship between dtPSA and the PET result (*p* = 0.002). The lower the dtPSA value, the higher the percentage of patients with a positive PET result.

Lesions detected in patients with different dtPSA values were shown to be statistically significantly different in SUVmax measurements (*p* = 0.030). Patients with a dtPSA under ix months have, on average, significantly higher SUVmax values (median 4.45) than patients with a dtPSA greater than six months (median 1.27), *p* = 0.031.

### 3.1. Local Recurrence

Local recurrences were detected in 13 patients (29% of positive patients). This accounted for 24% of the detected lesions. 

**Figure 1:**
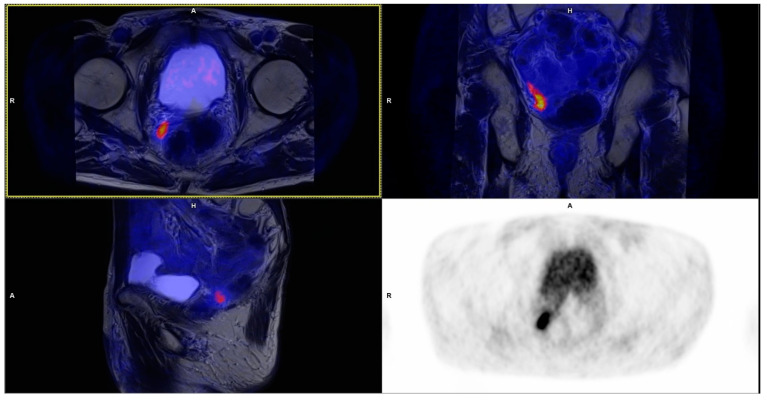
Local recurrence detected in patient with serum PSA level 0.22 ng/mL. The lesion is localized directly to the rectal wall. T2_tse_bl MR images fused with PET (upper images and lower left) and PET AC images (lower right).

**Figure 2:**
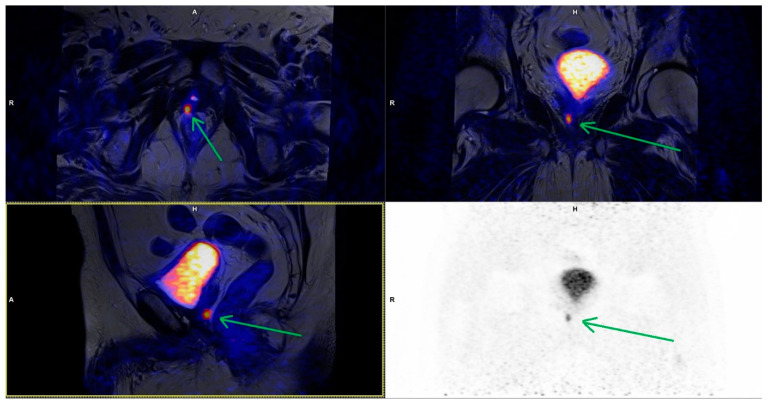
Local recurrence detected in patient with serum PSA level 0.27 ng/mL. The lesion is located to the right of the subvesical part of the urethra (green arrow). T2_tse_bl MR images fused with PET (upper images and lower left) and PET AC images (lower right).

All lesions were characterized by high focal PSMA uptake (Figure 1); in eight of them abnormalities of the MR images were found, corresponding to local recurrence (PSMA-RADS-5). In two patients, the described lesions spread to the colon wall. In the case of other lesions, no involvement of adjacent organs was observed; they were limited to the prostatic bed (Figure 2).

### 3.2. Lymph Nodes

Thirty-five PSMA-positive lymph nodes were detected in 27 patients. Lymph node metastases accounted for 66% of detected recurrences, in 60% of positive patients.

Only one extrapelvic lymph node—left supraclavicular (metastatic origin confirmed in FNAB)—was detected. All other PSMA-positive lymph nodes were localized in the pelvis.

The small axis of lymph nodes was in the range of 3–11 mm. Only five of them were rated as PSMA-RADS-5.

Six PSMA-positive pelvic lymph nodes, considered metastatic in the final report, were not visualized in whole-body PET/MR, but only in PET/MR of the pelvis, which is performed immediately after whole-body acquisition and may be considered as delayed examination. In pelvic PET/MR, the PET acquisition time is longer than in the whole-body method.

### 3.3. Bone Metastases

**Figure 3:**
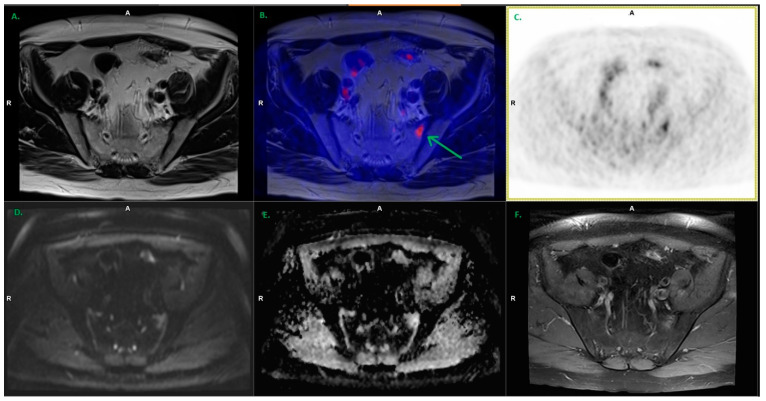
Moderate increased focal uptake of 18F-PSMA-1007 visible in the left ala of the sacrum (green arrow). Lesion considered benign based on appearance on MR images. In control PET/MR, performed in six months without any treatment, there was no evidence of increased PSMA uptake. ((**A**). T2_tse_tra, (**B**). Fused T2_tse_tra with PET AC, (**C**). PET AC, (**D**). DWI, (**E**). ADC map, (**F**). T1_vibe_CM—image after contrast agents’ injection).

In total, 17 PSMA-positive lesions in the skeletal system were detected in all patients (Figure 3). According to the PSMA-RADS criteria, only five were considered metastatic lesions (four PSMA-RADS-5 and one PSMA-RADS-4). All of them were verified and confirmed in the follow-up and other imaging methods.

Five PSMA-positive metastatic bone lesions were detected in three patients, which accounted for 9% of the detected lesions in 7% of positive patients.

The bone metastases were localized in the pelvis, femur, sternum and one rib.

Of the abovementioned patients, some had metastases in more than one organ. Local recurrence and a single metastatic lymph node were found in three patients, and metastatic lymph nodes and bone lesions were detected in three patients.

### 3.4. Patients’ Follow-Up

Only lesions in categories 4 and 5 according to PSMA-RADS were taken as PC foci, which were additionally verified during the 12-month follow-up of patients.

Eighteen patients with local recurrence or involved pelvic lymph nodes were preliminarily qualified for stereotactic radiotherapy. Finally, only 16 received this kind of treatment. After precise analysis of the obtained PET/MR images of the pelvis, two patients were disqualified because of localization of recurrence lesion direct to rectal wall. In those cases, the specialists’ board indicated the great advantages of the PET/MR method, which fuses the high-resolution multiparametric MR images of the prostatic bed with PSMA PET images, which enables individualization of treatment method in each patient (Figure 1). In all 16 patients, a decrease in PSA level in follow-up were observed. 

Ten patients were treated with radiotherapy in the pelvic area also with a decrease of PSA level in follow-up. 

Ten patients (and additionally two disqualified from stereotactic radiotherapy) were qualified for hormonotherapy. 

In addition, seven patients were observed without treatment (according to the specialists’ board decision).

18F-PSMA-1007 PET/MR and the obtained results influenced the clinical decision in 38 patients, which is 63% of the study group.

## 4. Discussion

The sensitivity and specificity of PSMA PET in BCR is significantly higher than other diagnostic imaging techniques [3,27,34,35,36,37,38,39,40,41].

The EAU indicates the 68Ga-PSMA-11 PET/CT as the standard of care in the diagnosis of recurrence in patients with BCR, and its performance is recommended from a PSA level > 0.2 [3,4].

The percentage of patients who are PSMA PET-positive correlates closely with serum PSA concentration, dtPSA and PSA velocity [42].

It should be noted that many studies show that a positive result of PSMA PET is being more dependent on a short dtPSA than on the PSA level itself, and dtPSA should be a factor indicating the need for PSMA PET, especially in patients with a low PSA <0.2 [42].

The results of our study confirm the previous observations. In the group of patients with a low PSA <0.5, a positive test result does not correlate with the PSA level, which is probably due to the small range of PSA levels and the small number of patients. However, there is a clear dependence of a positive result on dtPSA. Patients with early BCR and dtPSA < 6 months have a localized focus of recurrence significantly more often than patients with longer dtPSA.

Overall, in our study, the detection rate was 75%, shown at a PSA level of 0.1–0.5 ng/mL.

Currently, data from the literature indicate that the detection rates for PSA < 0.5 ng/mL are approximately 38–73% for 68Ga-PSMA-11 [43,44] and approximately 49–86% for 18F-PSMA-1007 [45,46,47,48,49]. There were suggestions indicating a high potential of 18F-PSMA-1007 in detecting, especially, early recurrence, even at a very low PSA value < 0.2ng/mL [45]. The application of 68Ga-PSMA-11 in patients with early BCR is also being analyzed, and the results in some publications are also very promising [50].

Eiber et al. reported that in 68Ga-PSMA-11 PET/CT a detection rate of 57.9% for PSA concentrations 0.2 is <0.5 ng/mL [16]. In analogous ranges of PSA level, Fendler et al. found a detection rate of 38% [42].

A review of sixteen articles involving 1309 patients showed a positive 68Ga-PSMA PET result in 42% and 58%, according to PSA categories 0–0.2, 0.2–1 ng/mL, respectively [44].

Available publications, although few, indicate some differences in the results obtained using 18F-PSMA-1007 and other radiotracers, but they are not statistically significant. However, the greater value of 18F-PSMA-1007 is emphasized, especially in the assessment of local recurrence, which results in a higher contrast between the recurrence focus and the tissues in its immediate location, such as the bladder and urethra [20]. In our study, we saw the benefits of this feature of 18F-PSMA-1007 (Figure 2), although there was no head-to-head comparison to PET/CT scans. 

In a publication from 2019 including a large group of 251 patients with BCR, 18F-PSMA-1007 PET/CT showed an average detection rate of 81.3%, and in a group of PSA 0.2 to <0.5 ng/mL, recurrence was found in 61.5%. The authors indicate a higher sensitivity of the technique compared to 68Ga-PSMA-11 PET [45].

Rahbar et al., evaluating the clinical value of the 18F-PSMA-1007 PET/CT technique in a study covering 100 pts, obtained very promising results; the evidence of recurrence was 86–100% for the relevant PSA ranges. The authors point out the particularly high detection rate in the group with early BCR, higher than in our study, amounting to 86% at PSA < 0.5, and indicate this group of respondents as a potential target with a significant treatment impact [46].

The results of other meta-analyses were not so optimistic. In the publication from 2019, the obtained results showed a 49% detection rate for PSA < 0.5 ng/mL vs. 86% with PSA ≥ 0.5; however, the authors emphasize the superiority of F-18-labeled ligands over Ga-68-labeled PSMA, especially in early relapse [49].

On the other hand, some authors in their reports analyze not only the type of radiotracer used, but also the acquisition technique, with an indication of the superiority of PET/MR, especially in patients with pelvic recurrence, also at very low PSA levels, regardless of the applied PSMA ligand [27,28,51,52,53,54].

Kranzbühler et al., using 68Ga-PSMA-11 PET/MR, showed a detection rate of 54.5%, of which it was 65% in patients with a PSA level of 0.2–0.5 ng/mL and 38.5% in patients with a PSA level less than 0.2 ng/mL [52].

A review of the literature published in Urology in 2019 is very important in terms of comparing the PET/CT and PET/MR techniques. The authors point to the high value of the examination in patients with BCR, enabling the detection of the recurrence site in patients with a PSA of 0.2–2 ng/ ml and even <0.2 ng/mL, especially in PET/MRI. They also indicate a constantly unsatisfactory number of studies performed regarding the comparison of the PET/CT technique with PET/MR [27].

Many authors indicate PSMA PET/MR as an examination during which a very sensitive and specific mpMR of the pelvis is performed at the same time, helpful in the detection of local recurrence and wbMR, with confirmed clinical value in the diagnosis of bone metastases [55].

In 2017, a report was published assessing the clinical value of 68Ga-PSMA-11 PET/MRI in BCR. A retrospective evaluation of PSMA PET/MR results in 56 patients with a mean PSA value of 0.99 ng/mL indicates a “detection rate” of 78.6%. In patients with PSA < 0.2 ng/mL, the recurrence site was localized in 44% of patients, with PSA 0.2–0.5 in 73%, for PSA values of 0.5–2 ng/mL, the “detection rate” was 80% [53].

The advantages of PET/MR over PET/CT in BCR stem primarily from the precise assessment of the pelvis in connection with high-resolution MR images, performed with the PI-RADS protocol, simultaneously with PET. Secondly, due to the longer duration of the examination, determined by the duration of MR, the PET acquisition time in most centers is longer that than in the routine PET/CT acquisition. So, we can talk about greater sensitivity and resolution of this technique by obtaining a larger number of counts. This is confirmed by scientific reports, especially in the few comparative PET/CT and PET/MR studies. In addition, in whole-body imaging, the multimodality, the combination of PET imaging with various MR images and sequences, including those diffusion-weighted images (DWI), is indicated as a great advantage of the technique, giving a synergistic effect enabling a more precise assessment of the images obtained [28,51,52,53,54,55].

It may seem surprising that 18F-PSMA-1007 PET/MR detected local recurrence in 13 patients with a negative previous mpMR result, and additionally, 8 of them were described as PSMA-RADS-5. The sensitivity and specificity of the 18F-PSMA-1007 PET method allows visualization of even very small or irregular lesions that are difficult to detect in mpMR, most likely due to the presence of postoperative lesions. Only the cooperation of two specialists and the evaluation of precise PET and MR images taken simultaneously allows the benefit from the synergistic effect of the PET/MR technique.

On the other hand, the duration of the PET/MR examination may negatively affect the patient’s comfort and in some cases may be a significant problem.

The currently introduced digital PET/CT, including PET/CT with long-axial field-of-view or whole-body PET/CT scanners, allows to perform a more sensitive examination in a much shorter time, but it should be remembered that they will not provide a multiparameter and thus precise images of the pelvic anatomy, which only mpMR can currently offer.

In one of the analyses comparing all available diagnostic techniques in patients with BCR, the authors emphasize the highest value of PET/MR as a method combining PET, whole-body MRI and multiparametric MRI in one study [27].

In 2019, a systematic review on the management of patients with oligometastatic disease was published in the World Journal of Urology. It emphasizes that it is necessary to introduce new diagnostic methods such as PSMA PET and DWI-MR [56], which in the case of PET/MR are performed during a one-time study. A very important publication from 2019, as one of the few, compared the value of PSMA PET/MR with PSMA PET/CT in patients with biochemical recurrence of PC. Examination with both methods was performed in each patient during one day. It was found that PET/CT and PET/MRI have comparable value in the assessment of distant metastases, while PET/MR has a higher value in the case of local recurrence [28].

When analyzing the benefits of 18F-PSMA-1007 PET, the advantages of a radiotracer enable a more precise assessment of local recurrence or other lesions in the pelvis, which has been proven in the available reports. It should be noted, however, that the foci of recurrence/metastases, also at early BCR, are not always located in the pelvis. Kranzbühler et al., in their study, showed that even at the low PSA values, only 12.1% of patients had exclusive local recurrence [52]. In 39.4%, PSMA-positive lesions were located outside a standard salvage radiotherapy volume. In another study, in 42.8% of BCR patients with mean PSA level 3.5 ng/mL, PSMA-positive lesions were limited to the pelvis [42].

It is obvious that the risk of bone metastases rises with an increase in PSA level [3,4].

In assessing the clinical usefulness of 18F-PSMA-1007 in extrapelvic extent of recurrent disease, the diagnostic accuracy of the technique in assessing bone metastases cannot be overlooked. After the first very enthusiastic data indicating the numerous advantages of using this radiotracer, critical opinions appeared regarding the possibility of false-positive results regarding bone metastases. This is related to the accumulation of the tracer in NSBL, in benign changes of various etiologies, degenerative or post-traumatic, and other nonmalignant bone lesions [25,26].

Initially, reports showing a lower specificity of 18F-labeled tracers in the assessment of PSMA-positive foci in the skeletal system suggested that the presence of free fluorine is the cause of this state. Subsequent studies conducted with a thorough analysis of the preparation of 18F-PSMA-1007, in accordance with GMP and quality control, undermine this theory and the presence of free fluoride as the main cause of nonspecific tracer accumulation in benign bone lesions [57].

Arnfield et al. indicate that more than 43% of patients who underwent 18F-PSMA-1007 PET/CT for BCR had at least one PSMA-positive NSBL [25]. In a study comparing 18F-PSMA-1007 and 68Ga-PSMA-11 PET/CT scans, not head-to-head but matched corresponding patients due to various clinical variables, the number of NSBL in 18F-PSMA-1007 patients was significant higher than in 68Ga-PSMA-11 [48]. However, the authors of both publications show that the use of PSMA-RADS as a lesion assessment system with specific SUVmax analysis may be the key to the correct differentiation between benign and malignant lesions. 

Regarding bone lesions, the advantages of the PET/MR technique and the synergistic effect of using many diagnostic MR images, including DWI with simultaneous PET acquisition, should be pointed out again.

In our study, 17 PSMA-positive bone foci were found, but only five were considered metastatic lesions (only foci with evident anatomical correspondence or high focal uptake and lack of corresponding benign lesions). In this case, additional verification in MR images, including the DWI sequence and contrast enhancement in the pelvis, was very helpful (Figure 2). Although there was no histopathological verification of the reported lesions, further observation and follow-up examinations seem to confirm the above results. Sawicki et al. compared the diagnostic value of PSMA PET/CT vs. whole-body MR in the assessment of the staging of metastatic lesions in patients with BCR, and clinically significant superiority of PSMA PET/CT in comparison to whole-body MR in BCR was demonstrated [58]. However, the synergism resulting from the use of both techniques at the same time can be very helpful in differentiating lesions, especially PSMA-positive NSBL. 

Once more, it should be mentioned that our study of PET/MR of the pelvis performed after the whole-body PET/MR scan showed more PSMA-positive lesions than the whole-body examination. Three factors may contribute to this. First, pelvic PET/MR is a delayed examination relative to the whole-body acquisition. Secondly, the PET counting time in pelvic PET/MR is longer and it was 10 min per examination compared to 3 min per bed in the whole-body scan. Thirdly, in this part of the examination, a precise mpMR of the pelvis is performed, with dedicated MR sequences, MR images with higher resolution and higher contrast of soft tissues are obtained. In further studies, we plan to perform delayed pelvic examinations using the list-mode technique, thanks to which it will be able to observe PET images of the pelvis with counting time of both 3 and 10 min and compare them with each other.

In the study cited above, showing a high percentage of recurrences detected in 18F-PSMA-1007 PET/CT even at low PSA, the acquisition was started at 120 min after the injection of the radiotracer [43]. This may also have contributed to such good results. In our study, the acquisition started 80 min after the administration of the radiotracer, but PET/MR of the pelvis can be treated as delayed examinations, but they concern the pelvis itself, which could have a positive impact on the sensitivity of the technique for detecting lesions in the pelvis (prostate bed, regional lymph nodes and bones).

Rahbar’s research team in another study describes the results of comparing images obtained 60 and 120 min after the administration of the tracer, showing the benefits of acquisition after a longer time after the administration of the radioisotope [24].

In our study, the performance of 18F-PSMA-1007 PET/MR resulted in the modification of therapeutic procedures in 68% of patients. The most important group consisted of patients qualified for radiotherapy. High-resolution PET/MR images of the pelvis enabled precise qualification and treatment planning. Radiotherapists indicate great clinical benefits resulting from the possibility of using PSMA PET/MR images of the pelvis. Unfortunately, the technique is not available for routine use due to the lack of reimbursement and high costs.

And finally, because of the lower dose of ionizing radiation in comparison with PET/CT, PET/MR examination seems to be a very beneficial solution especially in relatively younger patients and patients with curative-intent treatment. Because of long expected survival, it is very important to minimalize cumulative irradiation dose. The PET/MR technique allows to reduce the radiation dose up to 70% per one examination in comparison with a PET/CT scan, but also, innovative solutions in new digital PET/CT scanners allows to reduce the ionizing radiation doses.

Of course, the limitations of our study include the lack of head-to-head comparison with the PET/CT technique or with a scan performed with 68Ga—PSMA-11, which resulted from the limited budget of the project; therefore, the results are compared with data from the literature. The absence of histopathological verification of all PSMA-positive foci could be treated as the other weak point of this study; therefore, the 12-month follow-up and verification in other laboratory and imaging tests were implemented. 

## 5. Conclusions

Results of our study and a review of the literature indicate that 18F-PSMA-1007 PET/MR can be an excellent diagnostic tool in patients with early BCR with very low PSA levels (0.1–0.5 ng/mL). The synergistic effect of combining 18F-labeled PSMA and whole-body PET/MR with precise multiparametric assessment of pelvic lesions is of particular benefit to patients with early BCR, when recurrent PC is locally advanced, before it comes to the spread of disease and increase of PSA level. It enables optimization of the treatment method and performing curative-intent therapy. 18F-PSMA PET/MR should be considered even in patients with very low PSA (also under 0.2 ng/mL), but it is very important to take dtPSA into account. However, to indicate the superiority of 18F-PSMA-1007 over 68Ga- PSMA-11 or PET/MR over the PET/CT technique, comparative head-to-head studies should be performed.

## Figures and Tables

**Table 1:** Patients with positive and negative PSMA scans regarding GS and dtPSA.

	PSMA-Positive Scans	PSMA-Negative Scans
Patients’ Number	45	15
GS < 7	4	6
GS = 7	28	7
GS > 7	13	2
dtPSA ≤ 6 months	34	4
dtPSA > 6 months	11	11

**Table 2:** Localization, mean SUVmax and size of detected PSMA-positive lesions.

Recurrence Site	No. of Detected Lesions	Mean SUVmax ± SD	Size Range (mm)
Prostatic bed	13	6.34 ± 2.53	6 × 4 up to 19 × 18 × 25
Pelvic lymph nodes	34	5.46 ± 3.28	3 × 3 up to 11 × 11
Extrapelvic lymph nodes	1 *	3.75	3 × 3
Bones	5	4.75 ± 1.82	5 × 4 up to 8 × 8

* FNAB confirmed.

## Data Availability

The data presented in this study are available on request from the corresponding author. The data are not publicly available due to ethical restrictions.
